# Long Non-Coding RNA *Myoparr* Regulates GDF5 Expression in Denervated Mouse Skeletal Muscle

**DOI:** 10.3390/ncrna5020033

**Published:** 2019-04-08

**Authors:** Keisuke Hitachi, Masashi Nakatani, Kunihiro Tsuchida

**Affiliations:** Division for Therapies against Intractable Diseases, Institute for Comprehensive Medical Science (ICMS), Fujita Health University, Toyoake 470-1192, Japan; hkeisuke@fujita-hu.ac.jp (K.H.); nakatani@fujita-hu.ac.jp (M.N.)

**Keywords:** promoter-associated long non-coding RNA, bone morphogenetic protein signaling, skeletal muscle atrophy, denervation, RNA-seq

## Abstract

Skeletal muscle is a highly plastic tissue and decreased skeletal muscle mass (muscle atrophy) results in deteriorated motor function and perturbed body homeostasis. *Myogenin* promoter-associated long non-coding RNA (lncRNA) *Myoparr* promotes skeletal muscle atrophy caused by surgical denervation; however, the precise molecular mechanism remains unclear. Here, we examined the downstream genes of *Myoparr* during muscle atrophy following denervation of tibialis anterior (TA) muscles in C57BL/6J mice. *Myoparr* knockdown affected the expression of 848 genes. Sixty-five of the genes differentially regulated by *Myoparr* knockdown coded secretory proteins. Among these 65 genes identified in *Myoparr*-depleted skeletal muscles after denervation, we focused on the increased expression of growth/differentiation factor 5 (GDF5), an inhibitor of muscle atrophy. *Myoparr* knockdown led to activated bone morphogenetic protein (BMP) signaling in denervated muscles, as indicated by the increased levels of phosphorylated Smad1/5/8. Our detailed evaluation of downstream genes of *Myoparr* also revealed that *Myoparr* regulated differential gene expression between myogenic differentiation and muscle atrophy. This is the first report demonstrating the in vivo role of *Myoparr* in regulating BMP signaling in denervated muscles. Therefore, lncRNAs that have inhibitory activity on BMP signaling may be putative therapeutic targets for skeletal muscle atrophy.

## 1. Introduction

Long non-coding RNAs (lncRNAs), which exceed 200 nucleotides in length, are derived from intergenic regions, intronic regions, and cis-regulatory regions (enhancers and promoters) [1], and show tissue-specific expression patterns compared to protein-coding RNAs [2,3]. Divergent species of lncRNAs have been identified from mammalian genomes, including the human genome. Although the nucleic acid sequences of lncRNAs among different species are poorly conserved, their biological function tends to be conserved between species, possibly because of their similar secondary structure [4].

Gain-of-function and loss-of-function of lncRNAs in cell culture systems have revealed that lncRNAs exhibit multiple biological roles, such as epigenetic regulation, transcriptional regulation, translational regulation, and functioning as structural cores, among others [5,6]. Correlations between mutations and dysregulation of lncRNAs and human diseases have been identified [7,8,9]. In addition, in vivo functions of lncRNAs have been established in dosage compensation and genome imprinting processes [10,11,12,13]. Other in vivo functions of several lncRNAs have been experimentally demonstrated using knockout mice. For instance, *Fendrr* regulates heart and body wall development [14] and *Neat1* regulates corpus luteum formation and pregnancy [15]. Furthermore, *Hotair* is essential for repression of *Hoxd* expression in cultured cells [16], however, the in vivo role of *Hotair* remains controversial [17]. Several genetic studies have also raised questions about the roles of multiple lncRNAs in the development of mice [18,19,20]. Thus, in vivo analysis of the molecular function of lncRNAs is essential in order to reveal the physiological roles of lncRNAs in differentiation, development, and various diseases.

Skeletal muscle is a highly plastic tissue that is required not only for motion but also for maintaining body homeostasis. During embryonic development, the number of proliferating mononuclear myoblasts determines the size of future skeletal muscle tissue. As development progresses, myoblasts cease growing and undergo differentiation to form multinuclear myotubes. This process, which is called myogenesis, has been studied as a model system of cell differentiation. The *myogenin* gene product is one of the major transcriptional regulators in myogenesis [21]. We previously identified *Myoparr*, a lncRNA expressed in the opposite direction from the promoter region of *myogenin* [22]. *Myoparr* promotes myogenic differentiation through the activation of neighboring *myogenin* expression. Intriguingly, *Myoparr* expression also increases during skeletal muscle atrophy caused by denervation and promotes muscle atrophy in mice. While *Myoparr* activates the expression of *myogenin* in vivo, the exact molecular mechanism by which *Myoparr* promotes skeletal muscle atrophy caused by denervation remains to be elucidated.

In the current study, we aimed to examine changes in the expression profiles of genes in denervated tibialis anterior (TA) muscles of mice following *Myoparr* knockdown. *Myoparr* knockdown caused the upregulation or downregulation of hundreds of genes at an earlier time-point after denervation. We focused on 65 genes that coded for potential secretory proteins in which expression was altered by *Myoparr* knockdown, and examined the involvement of these genes in skeletal muscle atrophy. We showed the importance of elevated growth/differentiation factor 5 (GDF5) expression—which is also known as bone morphogenetic protein (BMP) 14 and an inhibitor of muscle atrophy in mice [23]—for preventing muscle atrophy in *Myoparr*-depleted muscles under denervation. Therefore, our findings reveal that *Myoparr* promotes skeletal muscle atrophy by repressing GDF5/BMP signaling in denervated skeletal muscles and may help identify putative therapeutic targets for preventing and treating skeletal muscle atrophy.

## 2. Results

### 2.1. Knockdown of Myoparr Affected Global Gene Expression in Denervated Mouse Skeletal Muscles

**Figure 1:**
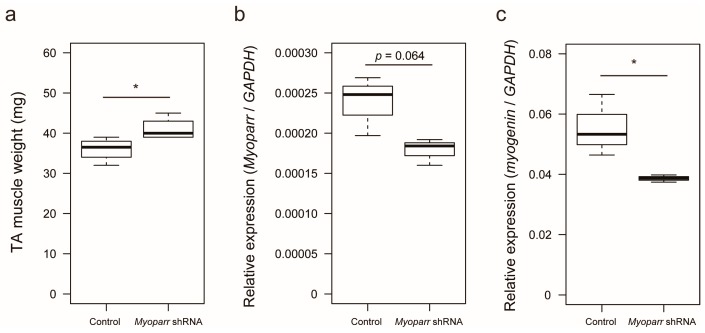
Knockdown of *Myoparr* attenuated skeletal muscle atrophy caused by denervation. (**a**) Box-and-whisker plots showing the weights of denervated tibialis anterior (TA) muscles of C57BL/6J mice electroporated with either control short hairpin RNA (shRNA) against *Lac*Z or *Myoparr*-specific shRNA. TA muscle weights were measured 3 days post denervation (*n* = 4 per group). * *p* < 0.05, unpaired two-tailed Student’s *t*-test. Central black bar indicates median; lower and upper box limits are 25th and 75th percentiles, respectively; whiskers show maximum and minimum values. (**b**) The level of *Myoparr* expression in denervated TA muscles electroporated either with control or *Myoparr* shRNA (*n* = 3 per group) was measured by quantitative reverse transcription polymerase chain reaction (qRT–PCR). *p* = 0.064, unpaired two-tailed Student’s *t*-test. Data were normalized to glyceraldehyde-3-phosphate dehydrogenase (*GAPDH)* expression. (**c**) Quantitative reverse transcription–polymerase chain reaction (qRT–PCR) showing decreased *myogenin* expression by *Myoparr* knockdown 3 d post denervation (*n* = 3 per group). * *p* < 0.05, unpaired two-tailed Student’s t-test. Data were normalized to *GAPDH* expression.

We previously showed that small hairpin RNA (shRNA)-mediated knockdown of *Myoparr* in skeletal muscle decreases *myogenin* expression and attenuates skeletal muscle atrophy 7 days post sciatic nerve transection [22]. To identify the early response genes by *Myoparr* knockdown other than *myogenin*, we examined skeletal muscle mass at 3 days post denervation. Increased skeletal muscle mass was observed in response to *Myoparr* knockdown after denervation compared with that of the control side of the mice (Figure 1a). Thus, *Myoparr* knockdown attenuated muscle atrophy at an earlier time point than previously reported.

**Figure 2:**
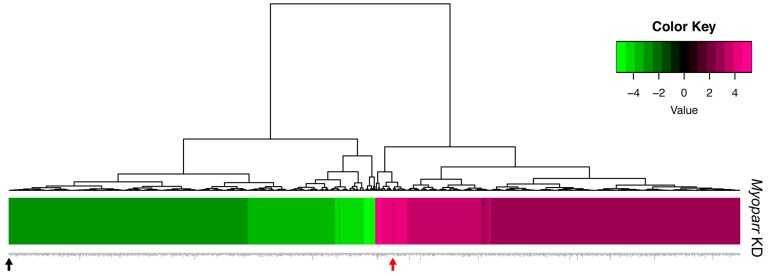
Knockdown of *Myoparr* altered global gene expressions in denervated skeletal muscle. The heatmap displayed the changes in expression for 848 genes altered by *Myoparr* knockdown in denervated TA muscle of C57BL/6J mice (values are z-scores). The black and red arrow indicate the decreased expression of *myogenin* and increased expression of *Gdf5*, respectively.

To better characterize the in vivo role of *Myoparr*, we examined global gene expression in *Myoparr*-depleted TA muscles using RNA-sequencing (RNA-seq) analysis. Tibialis anterior (TA) muscles were transfected with either *Myoparr*-specific or control shRNA and the sciatic nerve was then transected. Three days after denervation, the total RNA was extracted from the muscles and used for RNA-seq library construction. Before RNA-seq analysis, *Myoparr* expression was quantified by quantitative reverse transcription polymerase chain reaction (qRT–PCR). Although statistical significance (*p* < 0.05) was not observed, the level of *Myoparr* expression tended to decrease by *Myoparr* knockdown (Figure 1b). Instead, we observed that *Myoparr* knockdown significantly decreased *myogenin* expression, which we previously identified as a *Myoparr* target gene [22] (Figure 1c), indicating that electroporation-mediated *Myoparr* knockdown in TA muscles altered the expression of the downstream gene of *Myoparr*. RNA-seq analysis showed that knockdown of *Myoparr* affected global gene expression, although the rates of changes were relatively mild, possibly as a result of unaltered gene expression in the remaining untransfected myofibers. Therefore, we converted the log2 values of the RNA-seq results to z-score values. In this study, the z-score threshold was set to >2.5 or <−2.5. The gene expression dynamics in *Myoparr*-depleted muscles were shown by cluster analysis based on their z-score values. As illustrated by the heatmap in Figure 2, *Myoparr* knockdown altered the expression level of 848 genes in denervated TA muscles with 423 genes being upregulated and 425 genes being downregulated (Table S1). It is of note that the expression of the *myogenin* gene was also decreased as indicated by the black arrow in Figure 2. These results indicated that *Myoparr* knockdown globally affected gene expression in denervated muscles in mice.

### 2.2. Knockdown of Myoparr Increased the Expression Level of Growth/Differentiation Factor 5

The efficiency of transgene expression is approximately 30% of total myofibers at 3 days following electroporation [24], indicating that shRNA inhibits *Myoparr* expression in 30% of denervated TA muscle myofibers. However, we observed significant increases in weight of TA muscles following *Myoparr* knockdown. Based on these observations, we hypothesized that *Myoparr* knockdown altered the expression of secretory proteins and also affected the size of untransfected myofibers. To investigate this possibility, we screened the genes coding for secretory proteins among the 848 genes differentially regulated by *Myoparr* knockdown using ProteINSIDE analysis [25]. We identified genes coding for secretory proteins by screening for proteins that had been annotated as secretory proteins. The ProteINSIDE analysis extracted 65 genes with secreted annotation from the 848 genes regulated by *Myoparr* knockdown. Of these 65 genes, 18 were upregulated (Table 1) and 47 were downregulated (Table 2). Notably, RNA-seq data showed that *Myoparr* knockdown decreased the expression levels of *Col14a1*, *Dcn*, and *Sfrp1*, which are essential for muscle homeostasis and reported to increase following denervation [26,27,28]. This supported the notion that *Myoparr* knockdown attenuated muscle atrophy caused by denervation.

**Figure 3:**
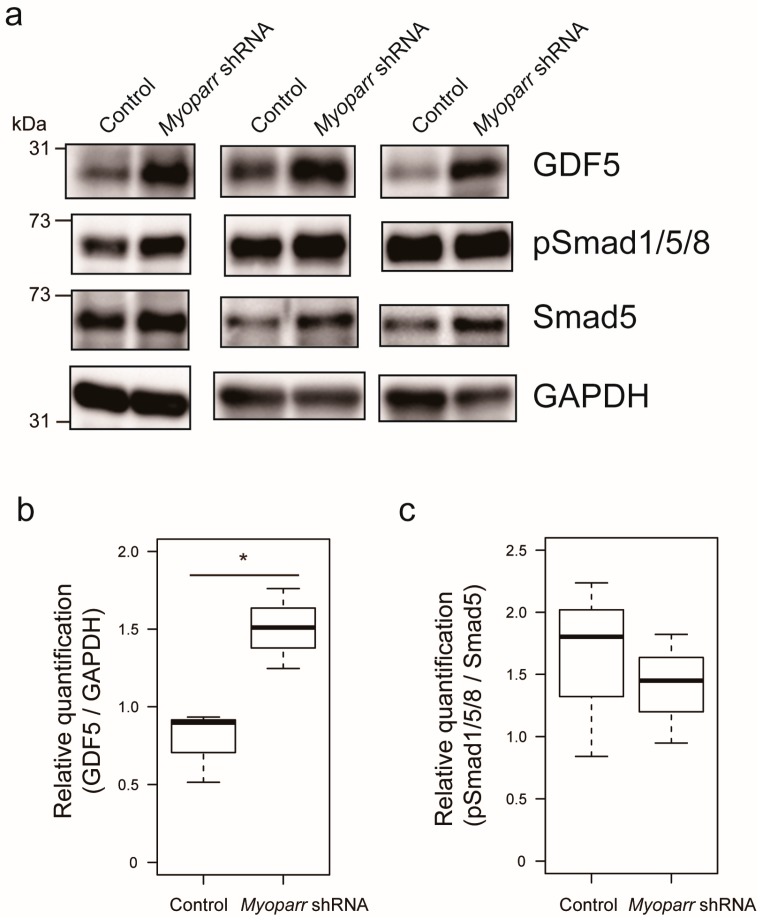
*Myoparr* knockdown activated bone morphogenetic protein (BMP) signaling through the expression of growth/differentiation factor 5 (GDF5) in denervated TA muscles of C57BL/6J mice. (**a**) Western blot showing increased expression of GDF5 by *Myoparr* knockdown in TA muscles 3 d post denervation. The activity of BMP signaling was indicated by the levels of phosphorylated Smad1/5/8 (pSmad1/5/8). GAPDH expression served as an internal control. Three independent experiments were shown. (**b**) Box-and-whisker plots showed relative quantification of GDF5 expression as GDF5/GAPDH ratio (*n* = 3 per group). * *p* < 0.05, unpaired two-tailed Student’s *t*-test. (**c**) Relative quantification of the activity of BMP signaling was shown as pSmad1/5/8/Smad5 ratio (*n* = 3 per group).

Among the various genes coding for secretory proteins, our attention was drawn to the *Gdf5* gene that has previously been shown to prevent muscle atrophy caused by denervation [23]. Thus, we examined the expression level of GDF5 in *Myoparr*-depleted TA muscles by western blot. At 3 days post denervation, *Myoparr* knockdown largely increased the expression level of GDF5 compared with that in the muscles transfected with control shRNA (Figure 3a,b). In agreement with the previous report that *Gdf5* is required for the activation of BMP signaling in denervated muscles [23], activated BMP signaling was also observed following *Myoparr* knockdown, as indicated by increased levels of phosphorylated Smad1/5/8 (Figure 3a). We also noticed that *Myoparr* knockdown increased the expression level of Smad5 (Figure 3a,c). Taken together, these results indicated that *Myoparr* knockdown prevented muscle atrophy after denervation by activating BMP signaling by increasing GDF5 and Smad5 expression.

### 2.3. Genes Regulated by Myoparr Knockdown Differed Between Myogenic Differentiation and Muscle Atrophy

**Figure 4:**
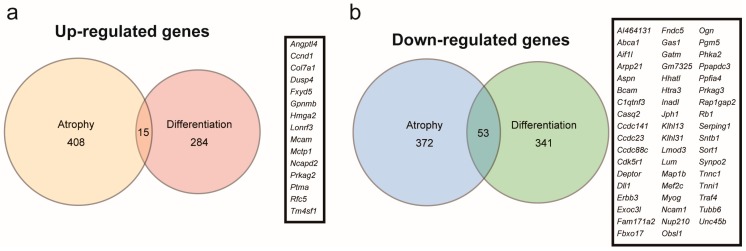
Comparison of genes commonly regulated by *Myoparr* knockdown between myogenic differentiation and muscle atrophy. (**a**) The intersection of genes upregulated by *Myoparr* knockdown in both skeletal muscle atrophy (423 genes) and myogenic differentiation (299 genes) showed low commonality. Gene symbols of the 15 commonly upregulated genes by *Myoparr* knockdown in both myogenic differentiation and muscle atrophy were shown in the panel. (**b**) The intersection of genes commonly downregulated by *Myoparr* knockdown in both skeletal muscle atrophy (425 genes) and myogenic differentiation (394 genes) also showed low commonality. Gene symbols of 53 commonly downregulated genes in both myogenic differentiation and muscle atrophy were shown in the panel.

*Myoparr* knockdown alters the expression of 693 genes in differentiating C2C12 cells, of which 299 are upregulated and 394 are downregulated [22]. The increased *Gdf5* expression in *Myoparr*-depleted skeletal muscles prompted us to compare the genes regulated by *Myoparr* knockdown in myogenic differentiation compared to those in muscle atrophy since the expression level of *Gdf5* is not changed in *Myoparr*-depleted C2C12 cells. We compared 423 upregulated and 425 downregulated genes in *Myoparr*-depleted TA muscles with 299 upregulated and 394 downregulated genes in *Myoparr*-depleted C2C12 cells, respectively (Figure 4a,b). Surprisingly, only 3.5% of the upregulated and 12% of the downregulated genes by *Myoparr* knockdown intersected between myogenic differentiation and muscle atrophy.

**Figure 5:**
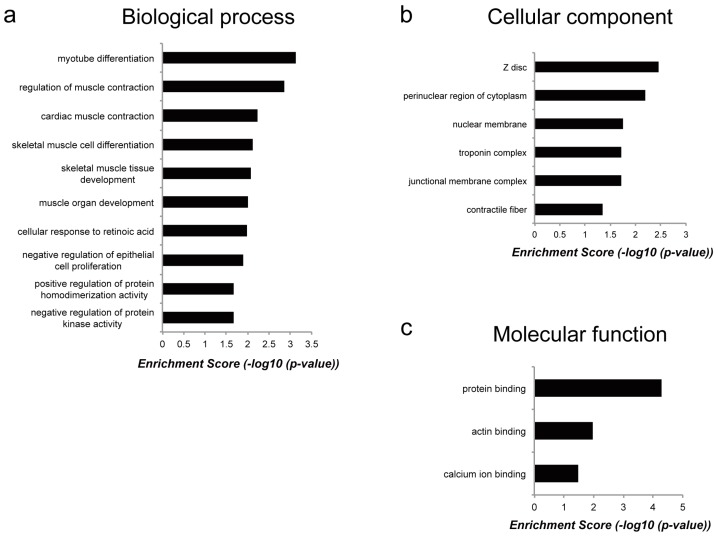
Classification of the annotated downstream genes of *Myoparr*. Genes commonly upregulated or downregulated by *Myoparr* knockdown in both myogenic differentiation and muscle atrophy were grouped into different functional gene ontology (GO) categories as indicated. (**a**) Biological process category. (**b**) Cellular component category. (**c**) Molecular function category.

To reveal the molecular functions of genes commonly regulated by *Myoparr* knockdown in both myogenic differentiation and muscle atrophy, we performed functional gene ontology (GO) enrichment analysis (Table S2). Although the enrichment score was not high, the results based on the biological processes category showed that genes regulated by *Myoparr* knockdown in both myogenic differentiation and muscle atrophy were enriched in skeletal muscle-related processes such as myotube differentiation, regulation of muscle contraction, and skeletal muscle cell differentiation (Figure 5a). It is of interest that these genes were also enriched in skeletal muscle-related categories of cellular component such as Z disc, troponin complex, and contractile fiber (Figure 5b) and of molecular function such as protein binding, including actin binding and calcium ion binding (Figure 5c). These results indicated that although genes commonly regulated by *Myoparr* knockdown both in vivo and in vitro were related to skeletal muscle function, most of downstream genes of *Myoparr* are different between myogenic differentiation and skeletal muscle atrophy.

## 3. Discussion

Multiple biological roles of lncRNAs have been revealed in vitro [5,6]; however, the roles of lncRNAs in vivo are still not well understood. We recently identified *Myoparr*, a promoter-associated lncRNA, and revealed its essential role in myogenic differentiation [22]. Interestingly, increased *Myoparr* expression promotes skeletal muscle atrophy in denervated skeletal muscles. *Myoparr* activates neighboring *myogenin* gene expression, which is one of the inducers of muscle atrophy [29]. However, the molecules expressed downstream of *Myoparr* during muscle atrophy have not been fully identified. In the current work, we investigated the downstream genes of *Myoparr* in denervated skeletal muscles in mice. Among hundreds of genes regulated by *Myoparr* knockdown, we focused on 65 genes that encoded secretory proteins. While myostatin, a cytokine belonging to the transforming growth factor-β (TGF-β) superfamily, is a pivotal inducer of muscle atrophy by increasing protein catabolism [30], BMP signaling can protect skeletal muscle mass after denervation [23,31]. In addition, skeletal muscle hypertrophy induced by myostatin inhibition is also dependent on activated BMP signaling [23,31]. We narrowed the analysis of the 65 *Myoparr*-regulated genes to target *Gdf5*, since *Gdf5* encodes a secretory protein belonging to BMP family and *Gdf5*-/- mice demonstrate aggravated muscle atrophy [23] that is accompanied with decreased BMP signaling activity. We largely observed increased GDF5 and Smad5 levels and activated BMP signaling in *Myoparr*-depleted skeletal muscles after denervation. It is noteworthy that the expression level of *Smad5* was not increased by *Myoparr* knockdown in RNA-seq analysis, suggesting that *Myoparr* represses Smad5 expression at the post-transcriptional level. Intriguingly, *Gdf5* expression in denervated muscles is independent of *myogenin* gene [32]. Therefore, our findings indicated that *Myoparr* promoted muscle atrophy in denervated muscles through the inhibition of BMP signaling by repressing *Gdf5* expression in a *myogenin*-independent manner.

In innervated muscle, the expression of *Gdf5* is repressed by the activity-regulated transcriptional co-repressors Dach2 and Hdac9/Mitr [32]. Surgical denervation reduces *Dach2* and *Hdac9* expression and activates BMP signaling through upregulated *Gdf5* expression [23,31,32]. Notably, Dach2 and Hdac9 also repress *myogenin* expression in innervated muscle through the minimal promoter region [33,34]. Although it is unclear whether Dach2 and Hdac9 repress *Myoparr* expression in innervated muscle, it has been established that *Myoparr* shares the minimal promoter region with *myogenin* in the myogenic differentiation process [22]. Thus, Dach2 and Hdac9 are common upstream repressors of *Gdf5*, *myogenin* and *Myoparr* expression in innervated muscle.

Our RNA-seq analysis of genes regulated by *Myoparr* knockdown in denervated muscles indicated that *Myoparr* regulated gene expression differently between in vivo muscle atrophy and in vitro myogenic differentiation. Although several studies have previously raised the importance of different physiological functions of lncRNA in vivo and in vitro [18,19,20], we speculate that the downstream genes of *Myoparr* may be determined depending on the cell status and context for the following reasons: (1) Composition of cells in skeletal muscle is largely different between muscle differentiation and muscle atrophy, as demonstrated by the different target genes of myogenin protein in each situation [29,35]. Thus, the myogenin-dependent function of *Myoparr* likely changes in the two situations; (2) different transcription factors are used in gene expression control between muscle differentiation and muscle atrophy, as shown by the different key regulators being MyoD or Dach2 in *myogenin* expression in both muscle differentiation and muscle atrophy [21,33]. *Myoparr* binds to Ddx17, a transcriptional co-activator of MyoD, and promotes the transcriptional activity of Ddx17 during myogenic differentiation [22]. Binding partner proteins influence the function of lncRNAs [36]. Thus, the utilization of different upstream regulators would change the *Myoparr*-binding protein and alter the molecular function of *Myoparr* in each situation. Taken together, our data suggested that the molecular function of lncRNAs was to be flexibly changeable depending on the gene expression profiles or their binding-proteins in vivo and in vitro.

In conclusion, we demonstrated for the first time that the expression of GDF5 was regulated by the *myogenin* promoter-associated lncRNA *Myoparr* in denervated skeletal muscle. Recently, it has become evident that lncRNAs are novel in vivo regulators of skeletal muscle mass [37,38,39,40,41,42,43] and muscle regeneration [38,44,45,46,47], each functioning through unique mechanisms. Intriguingly, Neppl et al. described the regulation of BMP signaling by lncRNA in skeletal muscle in which *Chronos* inhibits muscle growth by repressing *Bmp7* expression [48], indicating that several lncRNAs are pivotal negative regulators of BMP signaling in skeletal muscle. Therefore, lncRNAs that have inhibitory activity on BMP signaling such as *Myoparr* may be putative therapeutic targets for skeletal muscle atrophy. In addition to the role in myofibers, BMP signaling also plays an important role in the formation of blood vessels, essential components both in steady state of skeletal muscle and in the milieu of reinnervation of denervated skeletal muscle [32,49]. Collectively, examining the cell types which BMP signaling is activated by *Myoparr* knockdown in denervated skeletal muscle will further define the molecular roles of *Myoparr* in muscle atrophy.

## 4. Materials and Methods 

### 4.1. Animal Experiments

All mice used were male C57BL/6J strain, purchased from the Japan SLC (Shizuoka, Japan) and housed in cages with a constant temperature (24 °C) and a 12:12 h light-dark cycle. All animal experiments were conducted under protocols approved by the Institutional Animal Care and Use Committee of Fujita Health University. Prior to electroporation, the TA muscles of 8-week-old mice were injected with 30 μg endotoxin-free plasmid DNA containing either control shRNA against *Lac*Z or *Myoparr*-specific shRNA in a 30 μL saline solution using a 34-gauge needle from the ankle side to approximately 5 mm under anesthesia. The sequences of control shRNA and *Myoparr* shRNA are previously described [22]. Electric pulses (150 V/cm, 6 pulses, 50 ms pulses of 1-Hz frequency) were applied to the TA muscles with a tweezer-type electrode using a CUY21EDIT electroporator (Bex Co. LTD., Tokyo, Japan). A 3-mm fragment of the sciatic nerve was excised under anesthesia after electroporation. Three days after surgery, the mice were sacrificed and the TA muscles were collected, weighed, and processed for RNA and protein extraction.

### 4.2. RNA Isolation, Quantitative Reverse Transcription Polymerase Chain Reaction, Library Construction, Sequencing, and Data Analysis

Total RNA was extracted from the TA muscles and purified using an miRNeasy Mini kit with DNase I (QIAGEN, Hilden, Germany) according to the manufacturer’s protocol. One microgram of total RNA was used for the reverse transcription reaction using the SuperScript III First-Strand Synthesis System with random primers (Thermo Fisher Scientific, Waltham, MA, USA). The qRT–PCR was conducted using SYBR Premix Ex Taq (Takara, Shiga, Japan) according to the manufacturer’s protocol. Primers used for qRT–PCR were previously described [22].

One microgram of total RNA was used for purification of Poly(A)+ RNAs using the NEBNext Poly (A) mRNA Magnetic Isolation Module (New England Biolabs, Ipswich, MA, USA). RNA-seq libraries were constructed using the NEBNext Ultra RNA Library Prep Kit for Illumina (New England Biolabs) according to the manufacturer’s protocol and sequenced with 100-bp pair-end reads using an Illumina HiSeq 1500 system (Illumina, San Diego, CA, USA) at Fujita Health University. The average number of reads of each sample was approximately 5 million. The bcl2fastq 1.8.4 software was used for base calling. The RNA-seq raw data for each sample has been deposited in the DNA Data Bank of Japan (DDBJ) Sequence Read Archive under the Accession No. DRA007708. PRINSEQ ver. 0.20.4 software was used for quality trimming of row sequence data using the following command “-trim_qual_right 20 -min_len 30” [50]. Hisat2 ver. 2.0.5 software [51] was used for alignment of the trimmed reads to the mouse reference genome (mm10) applying the default parameters. The number of aligned reads was approximately 96% of the original reads. The aligned reads were converted and sorted to Bam files using SAMtools ver. 1.3.1 software [52] and the reads were counted with the HTSeq ver. 0.6.0 software [53] using the Mus_musculus_UCSC_mm10.gtf file and the following optional command: “--stranded=no --format=bam”. The value of the log2 fold change was calculated using DESeq2 ver. 1.12.4 software [54] and converted to a z-score using R software with the package Genefilter (https://bioconductor.org/packages/release/bioc/html/genefilter.html). Genes with z-score values >2.5 or <−2.5 were considered significant in this study. Gene ontology analysis was performed using DAVID ver. 6.8 (https://david.ncifcrf.gov/) and *p*-values < 0.05 were considered statistically significant.

### 4.3. Protein Extraction and Western Blot Analysis

Western blot analysis was performed as previously described in References [22,55]. In brief, the TA muscles were surgically isolated 3 d after denervation and frozen in liquid nitrogen. Samples were broken into pieces using a bead-homogenizer Shakeman2 (Bio Medical Science, Tokyo, Japan) and lysed in RIPA buffer consisting of 50 mM Tris-HCl (pH 8.0), 150 mM NaCl, 0.1% SDS, 1% Triton X-100, 0.5% sodium deoxycholate, protease inhibitors (1 mM phenylmethylsulfonyl fluoride, 1 μg/mL aprotinin, 4 μg/mL leupeptin), and phosphatase inhibitors (5 mM NaF, 5 mM β-glycerophosphate, 1 mM Na_3_VO_4_). Equal amounts of protein quantified using a Pierce BCA Protein Assay Kit (Thermo Fisher Scientific) were subjected to western blot analysis. The primary antibodies used included the GDF5 antibody A-10 (sc-373744, Santa Cruz Biotechnology, Dallas, TX, USA), the Smad5 antibody YY-6 (sc-101151, Santa Cruz Biotechnology), a phospho-Smad1(Ser463/465)/5(Ser463/465)/8(Ser426/428) antibody (#9511, Cell Signaling Technology, Danvers, MA, USA), and a glyceraldehyde-3-phosphate dehydrogenase (GAPDH) antibody (#2118, Cell Signaling Technology). The horseradish peroxidase (HRP)-linked secondary antibodies included an anti-mouse IgG (#7076, Cell Signaling Technology) and an anti-rabbit IgG (#7074, Cell Signaling Technology) as appropriate. For the detection of Smad5 and phosphorylated Smad1/5/8, Can Get Signal Immunoreaction Enhancer Solution (Toyobo, Osaka, Japan) was used. Semi-quantification analysis of western blot images was conducted with ImageJ software ver. 2.0.0-rc-69.

### 4.4. Statistical Analysis

Data were analyzed by using unpaired two-tailed Student’s t-test. *p* < 0.05 was considered statistically significant.

## Figures and Tables

**Table 1:** Upregulated genes encoding secretory proteins following *Myoparr* knockdown in denervated tibialis anterior (TA) muscles of C57BL/6J mice.

Gene Symbol	Protein ID	Entrez ID	log2FoldChange	z-score
*Fabp5*	Q05816	16592	0.374131436	4.002252172
*Lgals3*	P16110	16854	0.373718827	3.997828189
*Gdf5*	P43027	14563	0.361753963	3.869541224
*Pf4*	Q9Z126	56744	0.352996303	3.775641818
*Timp1*	P12032	21857	0.34793676	3.721393528
*Csf1*	P07141	12977	0.3124471	3.340874304
*Cfp*	P11680	18636	0.293360391	3.136227099
*Pxdn*	Q3UQ28	69675	0.273907602	2.927654795
*Nrcam*	Q810U4	319504	0.273420533	2.922432453
*Thbs4*	Q9Z1T2	21828	0.273376805	2.921963603
*Gars*	Q9CZD3	353172	0.272354163	2.910998862
*Egfl7*	Q9QXT5	353156	0.269565054	2.881094106
*F7*	P70375	14068	0.258828912	2.765981467
*Angptl4*	Q9Z1P8	57875	0.25297163	2.70317984
*Emilin2*	Q8K482	246707	0.247003963	2.639194667
*Col7a1*	Q63870	12836	0.245381068	2.621794029
*Sdc4*	O35988	20971	0.244800608	2.615570352
*Cd9*	P40240	12527	0.244138059	2.608466518

**Table 2:** Downregulated genes encoding secretory proteins following *Myoparr* knockdown in denervated TA muscle of C57BL/6J mice.

Gene Symbol	Protein ID	Entrez ID	log2FoldChange	z-score
*Olfml2b*	Q3V1G4	320078	−0.481025783	−5.166721595
*Nid1*	P10493	18073	−0.449335456	−4.826938722
*Gpx3*	P46412	14778	−0.448922444	−4.822510417
*C3*	P01027	12266	−0.445172583	−4.782304504
*C1qtnf3*	Q9ES30	81799	−0.445104367	−4.781573094
*Srpx2*	Q8R054	68792	−0.436334685	−4.687544788
*Lifr*	P42703	16880	−0.429117336	−4.610160558
*Sorl1*	O88307	20660	−0.414098638	−4.449130463
*Col14a1*	Q80X19	12818	−0.386271994	−4.1507739
*Postn*	Q62009	50706	−0.370968623	−3.986691548
*Svep1*	A2AVA0	64817	−0.370316314	−3.979697508
*Pi16*	Q9ET66	74116	−0.367955107	−3.954380707
*Pltp*	P55065	18830	−0.364986609	−3.922552548
*Fndc5*	Q8K4Z2	384061	−0.362929143	−3.90049245
*F13a1*	Q8BH61	74145	−0.344575054	−3.703700378
*Dcn*	P28654	13179	−0.333557655	−3.585572109
*Hspg2*	Q05793	15530	−0.324036042	−3.483481619
*Sfrp1*	Q8C4U3	20377	−0.306352312	−3.293877119
*Cilp*	Q66K08	214425	−0.301045081	−3.236973125
*Serping1*	P97290	12258	−0.295270029	−3.175053165
*Lum*	P51885	17022	−0.293171855	−3.152556597
*Col19a1*	Q0VF58	12823	−0.28991971	−3.117687182
*Htra3*	Q9D236	78558	−0.288268162	−3.099979327
*Mfap4*	Q9D1H9	76293	−0.282983368	−3.043315901
*Aspn*	Q99MQ4	66695	−0.279272444	−3.003527469
*Ogn*	Q62000	18295	−0.277371455	−2.983145114
*Apoe*	P08226	11816	−0.276102919	−2.969543903
*Adamts2*	Q8C9W3	216725	−0.274377529	−2.951044316
*Islr*	Q6GU68	26968	−0.273173486	−2.938134598
*Nucb2*	P81117	53322	−0.272713326	−2.933200774
*Lama2*	Q60675	16773	−0.271718749	−2.922536945
*St3gal1*	P54751	20442	−0.262186218	−2.820329392
*Col1a1*	P11087	12842	−0.262170298	−2.820158698
*Hsd17b11*	Q9EQ06	114664	−0.261915887	−2.81743091
*Igsf10*	Q3V1M1	242050	−0.260696015	−2.804351474
*Cp*	Q61147	12870	−0.259206576	−2.788381747
*Gas6*	Q61592	14456	−0.256647441	−2.760942767
*Serpine1*	P22777	18787	−0.252661608	−2.718206768
*Serpine2*	Q07235	20720	−0.250764111	−2.697861854
*Comp*	Q9R0G6	12845	−0.246462973	−2.651745163
*Fgl2*	P12804	14190	−0.241340354	−2.596820573
*Lox*	P28301	16948	−0.240518253	−2.588006027
*Timp3*	P39876	21859	−0.240498779	−2.587797228
*Qsox1*	Q8BND5	104009	−0.239576044	−2.577903687
*Galnt1*	O08912	14423	−0.239367349	−2.575666064
*Cxcl14*	Q9WUQ5	57266	−0.238871098	−2.570345274
*Timp2*	P25785	21858	−0.234496758	−2.523443712

## Supplementary Materials

The following are available online at https://www.mdpi.com/2311-553X/5/2/33/s1, Table S1: Genes upregulated or downregulated by *Myoparr* knockdown in denervated tibialis anterior (TA) muscle of C57BL/6J mice, Table S2: Gene ontology (GO) annotation of genes commonly upregulated or downregulated by *Myoparr* knockdown for myogenic differentiation and muscle atrophy.
